# Glucose Metabolism and Glucose Transporters in Breast Cancer

**DOI:** 10.3389/fcell.2021.728759

**Published:** 2021-09-06

**Authors:** Eunah Shin, Ja Seung Koo

**Affiliations:** Department of Pathology, Yonsei University College of Medicine, Seoul, South Korea

**Keywords:** breast cancer, glucose metabolism, glucose transporter, pentose phosphate pathway, serine/glycine pathway

## Abstract

Breast cancer is the most common malignancy in women worldwide and is associated with high mortality rates despite the continuously advancing treatment strategies. Glucose is essential for cancer cell metabolism owing to the Warburg effect. During the process of glucose metabolism, various glycolytic metabolites, such as serine and glycine metabolites, are produced and other metabolic pathways, such as the pentose phosphate pathway (PPP), are associated with the process. Glucose is transported into the cell by glucose transporters, such as GLUT. Breast cancer shows high expressions of glucose metabolism-related enzymes and GLUT, which are also related to breast cancer prognosis. Triple negative breast cancer (TNBC), which is a high-grade breast cancer, is especially dependent on glucose metabolism. Breast cancer also harbors various stromal cells such as cancer-associated fibroblasts and immune cells as tumor microenvironment, and there exists a metabolic interaction between these stromal cells and breast cancer cells as explained by the reverse Warburg effect. Breast cancer is heterogeneous, and, consequently, its metabolic status is also diverse, which is especially affected by the molecular subtype, progression stage, and metastatic site. In this review, we will focus on glucose metabolism and glucose transporters in breast cancer, and we will additionally discuss their potential applications as cancer imaging tracers and treatment targets.

## Introduction

Breast cancer is the most common malignancy in women worldwide, and ranks top in the cause of death in female cancers worldwide (Bray et al., 2018). A total of 2.1 million women were newly diagnosed with breast cancer in 2018, and 627,000 women died of breast cancer (Bray et al., 2018). Breast cancer is increasing in underdeveloped and developing countries, and it is decreasing in developed countries since the early 2000s (Rossouw et al., 2002; Bray et al., 2004; DeSantis et al., 2015). Breast cancer presents with diverse characteristics. To categorize such diverse features of breast cancer, molecular subtypes have been developed: luminal A, luminal B, HER-2, and basal-like type. Moreover, estrogen receptor (ER), progesterone receptor (PR), and HER-2 are the main targets for targeted therapy in breast cancer, and samples/cases that are negative for these three receptors are defined as triple negative breast cancer (TNBC), which comprises about 15% of breast cancer cases. Each of the molecular subtypes of breast cancer and TNBC shows distinct clinical and molecular features in treatment response. Generally, breast cancer is treated with surgery, chemo-radiotherapy, and targeted therapy for biomarkers. Breast cancer showing hormone receptor expressions is treated with hormonal therapy such as tamoxifen, and breast cancer showing HER-2 amplification is treated with targeted therapy such as trastuzumab. Those that do not have any treatment targets are treated with a non-specific chemotherapy.

One of the fundamental characteristics of cancer cells that differs from normal cells is metabolic reprogramming—producing energy through glycolysis rather than mitochondrial oxidative phosphorylation, which is known as the Warburg effect after the German scientist Otto Warburg who first described it in the 1950s. The Warburg effect was first described in the 1950s by Otto Warburg, a German scientist, who stated that cancer cells secrete high levels of lactate because of an increase in glycolysis (Warburg, 1956). In the process of glycolysis, which is one of the main processes of glucose metabolism, glucose can enter cancer cells by glucose transporters. As a result, various glucose metabolites are produced that are related to diverse metabolic pathways, such as the serine/glycine metabolic pathway and pentose phosphate pathway (PPP). These glucose metabolic pathways and glucose transporters have pivotal roles in cancer metabolism as well as in cancer progression and metastasis, and such metabolic characteristics can be used in imaging diagnosis and targeted therapies. This review will focus on the glucose metabolic pathways, such as glycolysis, serine/glycine pathway, and PPP, in breast cancer and glucose transporters used in glycolysis and their potential implications in clinical practice.

## General Aspects of Glucose Metabolism and Related Metabolic Pathways in Cancer

**FIGURE 1:**
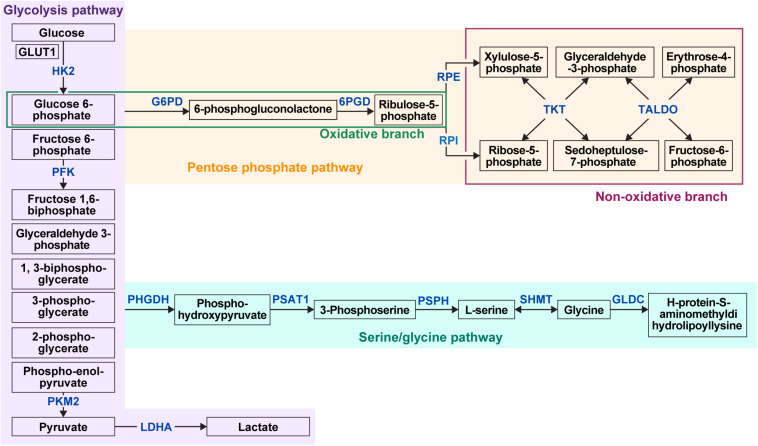
Overview of glucose metabolism in cancer cells. Glucose metabolism in tumor cells consists of three main types: glycolysis, the pentose phosphate pathway (PPP), and the serine/glycine pathway. First, in glycolysis, glucose influx occurs in the cell by glucose transporter GLUT1. Using HK2, PFK, and PKM2, glucose becomes pyruvate and is eventually converted to lactate by LDHA. PPP comprises an oxidative branch and a non-oxidative branch, where glucose 6-phosphate is converted to 6-phosphogluconolactone and then ribulose-5-phosphate by 6PGD and G6PD. The non-oxidative branch produces xylulose-5-phosphate by RPE and ribose-5-phosphate by RPI, and then produces fructose 6-phosphate, glyceraldehyde 3-phosphate, sedoheptulose- 7-phosphate, and erythrose-4-phosphate by TKT and TALDO through complex interchangeable reactions. The serine pathway starts with 3-phosphoglycerate, which is converted to phosphohydroxypyruvate by PHGDH, which is converted to 3-phosphoserine by PSAT1, and 3-phosphoserine is converted to serine by PSPH. In addition, glycine is converted by GLDC to H-protein-*S*-aminomethyldihydrolipoyllysine in glycine metabolism, which is linked to serine metabolism by SHMT in the form of reversible conversion. HK2, hexokinase II; PFK, phosphofructokinase; PKM2, pyruvate kinase isozymes M2; LDHA, lactate dehydrogenase A; G6PD, glucose 6-phosphate dehydrogenase; 6PGD, 6-phosphogluconate dehydrogenase; RPE, ribulose-5-phosphate epimerase; RPI, ribose 5-phosphate isomerase; TKT, transketolase; TALDO, transaldolase; PHGDH, phosphoglycerate dehydrogenase; PSAT1, phosphohydroxythreonine aminotransferase; PSPH, phosphoserine phosphatase; SHMT, serine hydroxymethyltransferase; GLDC, glycine decarboxylase.

Glucose metabolism consists of glycolysis and PPP, and glycolysis-related metabolic pathways consist of serine and glycine metabolism (Figure 1). A major pathway in the glucose metabolism of cancer cells is aerobic glycolysis, in the process of which glucose is first transported into the cancer cells by glucose transporters and then metabolized to pyruvate by various enzymes. Many enzymes are involved in this process, of which, the key enzymes are hexokinase II (HKII), phosphofructokinase (PFK), and pyruvate kinase (PK) (Li et al., 2015). Pyruvates produced in glycolysis are then moved into the mitochondria by mitochondrial pyruvate carriers 1 and 2, where they are turned into acetyl-CoA and oxaloacetate by pyruvate dehydrogenase and pyruvate carboxylase, respectively, to enter the TCA cycle for oxidative phosphorylation (OXPHOS) (Corbet and Feron, 2017). With one of the intermediate metabolites produced during the process of glycolysis, 3-phosphoglycerate (3PG), starts the serine pathway, in which 3-phosphoglycerate (3PG) is oxidized to 3-phosphohydroxypyruvate (pPYR) by phosphoglycerate dehydrogenase (PHGDH) and pPYR is transaminated to phosphoserine (pSER) by phosphoserine aminotransferase (PSAT). pSER is dephosphorylated to serine by phosphoserine phosphatase. In glycine metabolism, glycine is metabolized to H-protein-*S*-aminomethyldihydrolipoyllysine by glycine decarboxylase (GLDC), an important component of the glycine cleavage system. This serine metabolism and glycine metabolism are linked by serine hydroxymethyltransferse (SHMT), which causes a reversible conversion of serine and glycine (Locasale, 2013). Lastly, PPP is a metabolic pathway that occurs with glycolysis (Ramos-Martinez, 2017), playing a pivotal role in cell survival and growth by providing pentose phosphate for nucleic acid synthesis and also nicotinamide adenine dinucleotide phosphate (NADPH) for fatty acid synthesis and cell survival (Patra and Hay, 2014). PPP is comprised of two branches, the oxidative branch and non-oxidative branch. The oxidative branch converts glucose 6-phosphate (G6P) to ribulose-5-phosphate, CO_2_, and NADPH (Kruger and von Schaewen, 2003), and the non-oxidative branch produces glycolytic intermediates, such as fructose 6-phosphate (F6P), glyceraldehyde 3-phosphate (G3P), and sedoheptulose. These glycolytic intermediates are important for amino acid synthesis and produce ribose-5-phosphate (R5P) that is also important for nucleic acid synthesis (Stincone et al., 2015). Enzymes that are involved in the oxidative branch are 6-phosphogluconate dehydrogenase (6PGD) and glucose 6-phosphate dehydrogenase (G6PD), and those that are involved in the non-oxidative branch are ribulose-5-phosphate epimerase (RPE), ribose 5-phosphate isomerase (RPI), transaldolase (TALDO), and transketolase (TKT).

Cancer cells produce a high level of reactive oxygen species (ROS) compared to normal cells due to the increased activation of various metabolic pathways (Ahmad et al., 2005). Cancer cell metabolism is closely related to ROS homeostasis; they cause ROS detoxifications by using various substrates and metabolic intermediates in metabolic pathways, the most representative of which are glycolysis by the Warburg effect and PPP (Aykin-Burns et al., 2009). Glycolysis by the Warburg effect maintains redox homeostasis by being independent of mitochondrial OXPHOS that produces a large amount of ROS (Lee and Yoon, 2015), and PPP by producing ROS-detoxifying molecule, NADPH, by G6PD and 6-Phosphogluconate dehydrogenase (6PGDH) (Salazar, 2018).

Molecules involved in the regulation of glucose metabolism in cancer in general are oncogenes such as Ras, Src, and MYC, transcription factors such as hypoxia-inducible factor-1 (HIF-1), signaling pathway such as phosphoinositide 3-kinase (PI3K)/Akt/mammalian target of rapamycin (mTOR), and tumor suppressor such as p53. Oncogenes such as Ras, Src, and MYC increase the expression of HIF-1 that increases the expression of various glycolytic enzymes, and HIF-1, MYC, and KRAS increase glucose uptake by inducing GLUT expression. In addition, the PI3K/Akt/mTOR pathway induces glycolytic enzymes and GLUT expression, and p53 regulates glycolysis and GLUT through mTOR and AMP-activated protein kinase (AMPK) (Abdel-Wahab et al., 2019; Ghanavat et al., 2021).

## Glucose Transporters in Glycolysis

There are two families of glucose transporters: facilitative sugar transporters (GLUT, gene family name *SLC2A*) and Na^+^/glucose co-transporters (SGLT, gene family name solute carrier *SLC5A*). Additionally found families of glucose transporters are the Sugars Will Eventually be Exported Transporters (SWEET; SLC50) family and the Spinter protein (SLC63) family. SLC50 is a Na (+)/substrate co-transporter involved in the transport of glucose, myoinositol, and anions and located in the plasma membrane. SGLT1 (SLC5A1) and SGLT2 (SLC5A2) are important in glucose uptake with the former expressed mainly in the intestine and the latter in the kidney (Wright, 2013). GLUT has 14 isoforms that share structural features, such as 12 transmembrane domains, amino terminus, carboxy-terminus, and an *N*-glycosylation site. GLUTs can be subgrouped into three classes: class I (GLUT1–4 and GLUT14), class II (GLUT5, 7, 9, and 11), and class III (GLUT6, 8, 10, 12, and 13). Class I and class II GLUTs are called odd transporters, whereas class III GLUTs are called even transporters (Mueckler and Thorens, 2013). Except for GLUT13, which is a proton-driven myoinositol transporter, all GLUTs are facilitative transporters. These GLUT isoforms differ in the tissue type in which they are present, their location within the cells, cohesiveness with substrates, and control mechanism (Mueckler and Thorens, 2013). For instance, GLUT1 and GLUT3 are found in the brain, where they function mainly in glucose transport (Leino et al., 1997; Yeh et al., 2008), whereas GLUT3–5 and GLUT10–11 are found in the muscle (Bilan et al., 1992; McVie-Wylie et al., 2001; Rogers et al., 2002; Douard and Ferraris, 2008). Glucose is an important substrate for GLUT, but GLUT can also transport other substrates such as galactose, mannose, glucosamine, dehydroacetic acid, fructose, urate, and myo-inositol (Barron et al., 2016; Holman, 2020).

## Glucose Metabolism and Glycolysis-Related Metabolic Pathways in Breast Cancer

Cancer cells harbor a metabolic shift to aerobic glycolysis that plays an important role in tumor growth, progression, and metastasis; therefore, glucose metabolism and glycolysis-related metabolic pathways can have a diverse impact on cancer cells in breast cancer.

### Expression of Glycolysis-Related Enzymes and GLUTs in Breast Cancer

Breast cancer shows an increased expression of glycolysis-related enzymes, namely, HKII (Brown et al., 2002; Yang T. et al., 2018), 6-phosphofructo-2-kinase/fructose-2, 6-biphosphatase 3 (PFKFB3) (O’Neal et al., 2016), and pyruvate kinase M2 (PKM2) (Lin et al., 2015). In primary breast cancer, HKII is overexpressed in about 79% of tumors (Brown et al., 2002), which has been correlated with an increased histologic grade and proliferative activity (Sato-Tadano et al., 2013). The expression of 6-phosphofructo-2-kinase/fructose-2, 6-biphosphatase 3 activates PFK-1, a key enzyme in glycolysis (Okar et al., 2001), and is correlated with HER-2 expression and poor prognosis (O’Neal et al., 2016; Peng et al., 2018). Additionally, 6-phosphofructo-2-kinase/fructose-2, 6-biphosphatase 3 expression is related to the expression of vascular endothelial growth factor (VEGF)-α in breast cancer, which contributes to angiogenesis and distant metastasis (Peng et al., 2018). PFK-2 is a muscle isoform M2 of PK, a key enzyme in glycolysis, and its expression is correlated with a poor prognosis in breast cancer (Lin et al., 2015). Lactates produced by glycolysis are transported in and out of cells by monocarboxylate transporter (MCT) (Wilde et al., 2017). MCT1 overexpression in breast cancer is correlated with ER negativity, PR negativity, high Ki-67 labeling index (Li et al., 2018), basal-like type (Pinheiro et al., 2010), high grade, high stage, increased recurrence, and poor prognosis (Johnson et al., 2017). As for MCT4, tumoral MCT4 expression (Li et al., 2018) and stromal MCT4 expression (Baenke et al., 2015) are associated with poor prognosis.

Breast cancer has been reported to have an increased expression of GLUT1–6 and 12 (Table 1; Barron et al., 2016), and the most important glucose transporter for glucose uptake in breast cancer is GLUT1 (Grover-McKay et al., 1998; López-Lázaro, 2008; Furuta et al., 2010; Wuest et al., 2018). Glucose uptake by GLUT1 is important in the carcinomatous transformation and carcinogenesis of breast cancer, and it plays an important role in the early phase of breast cancer development (Young et al., 2011; Wellberg et al., 2016). GLUT1 overexpression in breast cancer is correlated with high histologic grade, high proliferative activity, poor differentiation, and poor prognosis (Pinheiro et al., 2011; Krzeslak et al., 2012). GLUT4 is an insulin-stimulated glucose transporter (Vargas et al., 2021), and glucose uptake is dependent on insulin stimulation in cancer cell lines (Harmon and Patel, 2004; Moreira et al., 2013; Guedes et al., 2016). It has also been reported that hyperinsulinemia increases the risk of breast cancer irrespective of the body mass index (BMI) (Lawlor et al., 2004; Kabat et al., 2009; Gunter et al., 2015), and so it can be postulated that insulin is associated with breast cancer. Cross-talks between signaling pathways regulated by 17 beta-estradiol (E2) and insulin-like growth factor (IGF) (Bruning et al., 1992; Conover et al., 1992), strong mitogen for cancer cells (Beckwith and Yee, 2014), and actions through ER-signaling (Katzenellenbogen and Norman, 1990) are some possible mechanisms associated with the insulin effect on breast cancer.

**TABLE 1:** GLUT expressed in breast cancer.

GLUT type	Patient number and diagnosis	Material and method	GLUT status	Related factors	References
GLUT1 GLUT2 GLUT3 GLUT4 GLUT5 GLUT6	33, IDC	IHC, FFPE	90.9% + + / + + + 90.9% + / + + 9.1% + 6.1% + 84.8% + / + + 50.0% +	n/a	Godoy et al., 2006
GLUT1	118, IBC	IHC, FFPE	42% positive	High Ki-67 High HG bcl-2 negative	Younes et al., 1995
GLUT1	124, IBC	IHC, FFPE	46% positive	High HG basal-like type PR negative High Ki-67	Pinheiro et al., 2011
GLUT1	100, IBC	IHC, FFPE	47% positive	High nuclear grade ER negative PR negative Shorter DFS, OS	Kang et al., 2002
GLUT1	78, IDC, No LN mets	IHC, FFPE	28.0% + in HG 1 63.8% + in HG 2 58.7% + in HG 3	n/a	Ravazoula et al., 2003
GLUT1	61, BC	IHC, FFPE	86.9% +	High HG	Alò et al., 2001
GLUT1	523, IBC −55 BLBC −231 non-BLBC	IHC, FFPE	76.4% + in BLBC 23.8% + in non-BLBC	High HG ER negative PR negative basal-marker + p53 expression	Hussein et al., 2011
GLUT1	132, TNBC	IHC, FFPE	65.2% + in tumor 5.3% + in stroma	n/a	Kim et al., 2013
GLUT1	276, IBC	IHC, FFPE	88.4% low 11.4% high	High HG ER negative PR negative No LN mets	Choi et al., 2013
GLUT1	809, IBC −692 IDC −114 ILC	IHC, FFPE	32.9% positive −37.3% + in IDC −6.1% + in ILC	High HG in ILC Shorter OS in ILC	Kim Y. H. et al., 2014
GLUT1 GLUT2	12, BC, 5, LN mets	IHC, FFPE	100% positive 100% positive	n/a	Brown and Wahl, 1993
GLUT1 GLUT3	70, BC	PCR, Western blotting	48.7% positive 21.0% positive	Higher HG	Krzeslak et al., 2012
GLUT1 GLUT4	30, BC	ICC	57% positive 43% positive	n/a	Binder et al., 1997
GLUT5	20, BC	IHC, FFPE	100% positive	n/a	Zamora-León et al., 1996
GLUT12	10, IBC	IHC, FFPE	80% positive	n/a	Rogers et al., 2003

*IDC, invasive ductal carcinoma; IHC, immunohistochemistry; ICC, immunocytochemistry; FFPE, formalin-fixed paraffin-embedded; IBC, invasive breast cancer; HG, histologic grade; ER, estrogen receptor; PR, progesterone receptor; LN, lymph node; BLBC, basal-like breast cancer; TNBC, triple negative breast cancer; ILC, invasive lobular carcinoma; PCR, polymerase chain reaction.*

**FIGURE 2:**
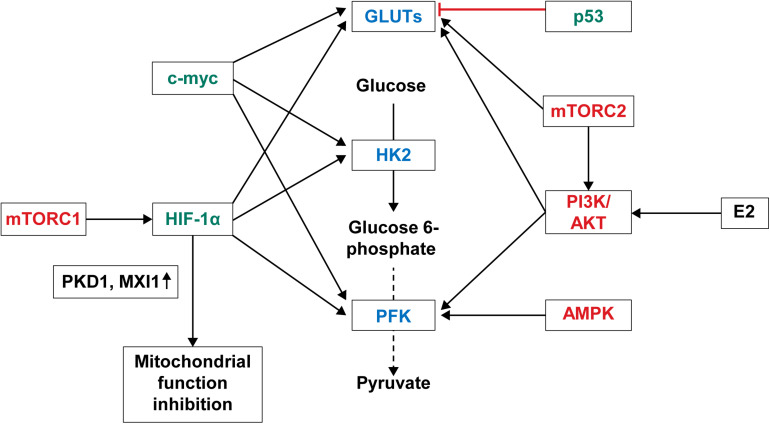
Regulation of glycolysis and glucose transporters in breast cancer. Important signaling pathways regulating glycolysis and glucose transporters in breast cancer are the PI3K/AKT, AMPK, and mTOR pathways. PI3K/AKT pathway activated by 17-estradiol (E2) or genetic mutations increases expression of PFK2 and GLUT. AMPK pathway activated in breast cancer transports GLUT4 to cell membrane through activation of PFK-2 and increases GLUT expression. mTORC1 among the mTOR complex increases the expression of PFK by activating HIF-1α. mTORC2 either activates AKT or increases GLUT1 expression. Transcription factors regulating glucose metabolism in breast cancer are c-myc, p53, and HIF-1α. As such, breast cancer with p53 mutation shows increased expression of GLUT because c-myc induces increased expression of GLUT, HK, and PFK, and p53 suppresses expression of GLUT. Lastly, activated HIF-1α increases expressions of HK, PFK, LDHA, and GLUT, and suppresses mitochondrial function by activating PKD1 and MXII. PI3K, phosphoinositide 3-kinase; AKT, Ak strain transforming protein kinase B; AMPK, AMP-activated protein kinase; mTOR, mechanistic target of rapamycins; PFK, phosphofructokinase; HIF, hypoxia-inducible factor; HK, hexokinase; LDHA, lactate dehydrogenase A; PKD1, pyruvate dehydrogenase kinase 1; MXI1, MAX interactor 1.

Overexpression of glycolysis-related enzymes and GLUTs in breast cancer is due to the activation of the signaling pathways controlling the enzyme expression in breast cancer (Figure 2). The main molecular pathways involved in the control of aerobic glycolysis are the PI3K/AKT, AMP-activated protein kinase (AMPK), mitogen-activated protein kinase, Wnt, and mTOR pathways (Engelman et al., 2006; Han et al., 2015; Cai et al., 2018; Hibdon et al., 2019; Irey et al., 2019). Among these, the PI3K/AKT, AMPK, and mTOR pathways are activated in breast cancer. PI3K/AKT activates phosphofructokinase-2 (PFK-2) by phosphorylation (Novellasdemunt et al., 2013; Lee et al., 2018). PI3K/AKT pathway activation leads to GLUT1 overexpression, which is then translocated from the cytoplasm to the plasma membrane (Samih et al., 2000). AKT is activated by E2, thus increasing the glucose uptake in MCF-7 breast cancer cell line through translocation of GLUT4 to the plasma membrane (Garrido et al., 2013). PIK3CA and AKT1 gene mutations are common in breast cancer (Castaneda et al., 2010; Koboldt et al., 2012), and PIK3CA mutation is usually found in ER-positive and HER-2 positive breast cancer. AMPK translocates GLUT4 to the cytoplasmic membrane by activating PFK-2 (Marsin et al., 2000) and increases GLUT1 expression (Barnes et al., 2002). AMPK is highly expressed in TNBC and known to be associated with poor prognosis (Huang et al., 2016). mTOR is a downstream effector of AKT, comprising mTOR complex 1 (mTORC1) and mTOR complex 2 (mTORC2) (Hara et al., 2002; Vivanco and Sawyers, 2002; Baretić and Williams, 2014). mTORC1 promotes the transition from OXPHOS to glycolysis and increases the expression of HIF-1α, which in turn increases the expression of glycolysis-related enzymes such as PFK (Düvel et al., 2010). mTORC2 promotes glycolysis by activating AKT (García-Martínez and Alessi, 2008; Ikenoue et al., 2008; Cybulski and Hall, 2009) and GLUT1-related glucose uptake (Beg et al., 2017). mTOR is activated in breast cancer through HER-2 overexpression, PI3K pathway alteration, and mTOR mutation (Hare and Harvey, 2017). Second, the increased expression of glycolysis-related enzymes in breast cancer is because of the activation of transcription factors (Figure 2). The transcription factors associated with glycolysis are c-myc, p53, and HIF-1. c-myc is responsible for increasing the gene expression of glycolysis-related genes and, consequently, glycolysis-related enzymes, such as GLUT, HK, and PFK (Hsieh et al., 2015). Moreover, estrogen is responsible for the increased expression of c-myc, and about 80% of breast cancers are ER-positive (Butt et al., 2008). p53 is a well-known tumor suppressor, gene mutations of which are found in most cancers including breast cancer. p53 mutation is found in about 20%–30% of breast cancers and more often in ER-negative breast cancer. p53 suppresses phosphoglycerate mutase (PGM), GLUT1, GLUT3, and GLUT4 expression (Kawauchi et al., 2008; Vousden and Ryan, 2009); hence, p53 mutation leads to an increased glycolysis in breast cancer. Lastly, the transcription factor HIF-1α, which is activated by hypoxia, is an important regulator in glycolysis and increases the expression of glycolysis-related molecules, such as HKII, PFK-1, lactate dehydrogenase (LDH) A, GLUT-1, and GLUT-3. HIF-1α promotes the metabolic shift to glycolysis by suppressing the mitochondrial function through the activation of pyruvate dehydrogenase kinase 1 (PKD1) and MAX interactor 1 (MXI1) (Denko, 2008). HIF-1α overexpression has been reported in breast cancer (Zhong et al., 1999), and it is attributed to the increased expression of glycolysis-related proteins in breast cancer because HIF-1α overexpression is related to HER-2 positivity (Giatromanolaki et al., 2004) and TNBC (Jin et al., 2016).

Breast cancer is susceptible to sex hormones such as estrogen, which may have an effect on the regulation of glucose metabolism. E2 and ERα stimulation activates the MAPK pathway (Ronda et al., 2010a, b), regulates expression of GLUT4 (Barros et al., 2006, 2008), and increases glucose uptake (Niu et al., 2003; Gorres et al., 2011). Furthermore, E2 activates the PI3K pathway that is involved in glucose metabolism in breast cancer cells (Simoncini et al., 2000; Lee et al., 2005), and suppresses phosphatase and tensin homolog (PTEN), a phosphatidylinositol-3 kinase inhibitory protein (Noh et al., 2011).

#### Expression of Glycolysis-Related Enzymes and GLUTs in TNBC

Triple negative breast cancer is defined as breast cancer that is negative for ER, PR, and HER-2 and accounts for about 15% of breast cancer cases. Basal-like breast cancer (BLBC) is defined as those that have high expressions of basal genes in gene expression studies such as DNA microarray. Therefore, TNBC and BLBC are not the same in the strict sense of definitions (Carey et al., 2010), although they can overlap in many instances. TNBC is a heterogeneous group, and many researches have focused on the subgrouping of TNBC. Lehmann et al. (2011) have grouped TNBC further into basal-like1, basal-like2, mesenchymal, and luminal androgen receptor, and Burstein et al. (2015) have grouped TNBC further into basal-like immune-activated, basal-like immune suppressed, mesenchymal, and luminal androgen receptor. The general characteristics of TNBC include the histological characteristics of high grade, high proposition index, and tumor necrosis, and clinical characteristics of higher rate of metastasis and poor prognosis (Kumar and Aggarwal, 2016; Borri and Granaglia, 2020). With these histological and clinical features, TNBC can be postulated to be of high metabolic status. One of the important metabolic features of TNBC is high glucose uptake, and GLUT1 overexpression is seen in TNBC (Hussein et al., 2011; Oh et al., 2017). High expression of glycolysis-related enzymes, such as HK2 (Jiang S. et al., 2012), PKM2 (Christofk et al., 2008; Ma et al., 2019), and LDH (McCleland et al., 2012; Huang et al., 2016; Dong et al., 2017), and that of lactate transporters MCT1 and MCT4 have also been reported in TNBC (Pinheiro et al., 2010; McCleland et al., 2012; Doyen et al., 2014). The high expression of glycolysis-related proteins in TNBC is owing to the fact that the glycolysis regulatory factors, such as HIF-1 (Lin et al., 2016; De Blasio et al., 2020), c-myc (Palaskas et al., 2011; Shen et al., 2015), and EGF signaling (Avanzato et al., 2018), are promoted in TNBC. Therefore, TNBC cells are much more dependent on glucose metabolism than non-TNBC cells (MCF-7) (Robey et al., 2005), and GLUT1 inhibition shows a more anti-proliferative effect for TNBC cells than non-TNBC cells (MCF-7) (Yang et al., 2021).

### Non-glycolysis Glucose Metabolism Pathway in Breast Cancer

In glucose metabolism, non-glycolysis metabolic pathways, such as the serine/glycine metabolic pathway and PPP, play important roles in breast cancer. The expression of serine/glycine metabolic pathway-related proteins in breast cancer differs depending on the breast cancer molecular subtype. Serine metabolic pathway-related proteins were highly expressed in TNBC (Labuschagne et al., 2014), and glycine metabolic pathway-related proteins were highly expressed in HER-2 type breast cancer (Kim S. K. et al., 2014). The basal-like type also showed a higher expression of serine/glycine metabolic pathway-related proteins among the TNBC subtypes (Noh et al., 2014). Analysis using the cBioPortal TCGA Pan-Cancer Atlas shows PHGDH amplification in approximately 2.2% of breast cancers (Geeraerts et al., 2021a). PHGDH expression is observed frequently in ER-negative breast cancer (Possemato et al., 2011), and increased PHGDH expression in breast cancer is associated with poor prognosis (Locasale et al., 2011; Possemato et al., 2011). Similarly, phosphoserine aminotransferase 1 (PSAT1) is more frequently expressed in ER-negative breast cancer and is associated with poor prognosis (Gao et al., 2017). Serine hydroxymethyltransferase 2 expression level is associated with the histologic grade of breast cancer (Yin, 2015).

High expression of PPP-related enzymes, such as 6PGD (Yang X. et al., 2018) and TKT (Benito et al., 2017; Yang X. et al., 2018), is reported in breast cancer. G6PD, one of the PPP-related enzymes, is associated with the molecular subtype of breast cancer, and G6PD overexpression is associated with poor prognosis of breast cancer (Pu et al., 2015; Dong et al., 2016). 6PGDH expression is high in TNBC, and the expression of G6PDH and 6PGL are high in HER-2 type (Choi et al., 2018b). The expression of G6PDH is also the highest in brain metastasis among metastatic breast cancers (Cha et al., 2017). The expression of TKT is associated with tumor size and high TKT expression is associated with poor prognosis in a mouse model of breast cancer (Tseng et al., 2018). Increased PPP flux by G6PD and HK2 enhancement induces tamoxifen resistance in breast cancer (Wang et al., 2016). An increase in HK2 transcription by the yes-associated protein (YAP) axis also promotes the migration of breast cancer cells (Tseng et al., 2018).

### Glucose Metabolism in the Tumor Microenvironment of Breast Cancer

**FIGURE 3:**
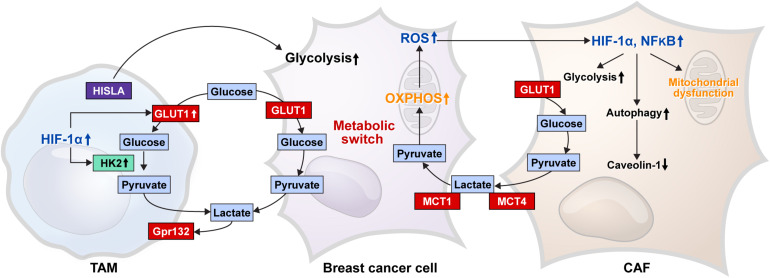
Glucose metabolic interaction between breast cancer cells and stromal cells. The glucose metabolic interaction between the breast cancer cell and CAF is presented as the reverse Warburg effect, where mitochondrial dysfunction results in a decrease in caveolin-1 levels because of increased autophagy, and an increase in glycolysis occurs by enhanced HIF-1α and NF-κB in CAF. Lactate produced by glycolysis is transferred to cancer cells by MCT4 in CAF and MCT1 in cancer cells, which is converted to pyruvate and used as a material for mitochondrial OXPHOS. ROS produced by the OXPHOS process cause an increase in HIF-1α and NF-κB in CAF. TAM, one of the immune cells of breast cancer stroma, shows increased glycolysis because of the increased GLUT1 and HK2 activity by enhanced HIF-1α expression; therefore, TAM can compete with cancer cells for glucose. G protein-coupled receptor 132 (Gpr132) senses the lactate produced by glycolysis to convert the macrophage to an M2-like phenotype, which promotes cancer cell adaptation, migration, and invasion. HIF-1α-stabilizing long non-coding RNA (HISLA) is transferred from TAM to breast cancer cells through extracellular vesicle transmission, and then, HISLA promotes glycolysis in breast cancer cells. Breast cancer cells have a metabolic switch that controls glycolysis and OXPHOS depending on the circumstances. CAF, cancer-associated fibroblast; HIF, hypoxia-inducible factor; MCT, monocarboxylate transporter; OXPHOS, oxidative phosphorylation; HK, hexokinase; ROS, reactive oxygen species; TAM, tumor-associated macrophage.

Breast cancer is one of those tumors that harbors tumor stroma, the main cell components of which include cancer-associated fibroblasts (CAFs), cancer-associated adipocytes (CAAs), and immune cells. These stromal cells affect the development, progression, and metastasis of breast cancer through various interactions with breast cancer cells (Mao et al., 2013; Soysal et al., 2015; Choi et al., 2018a; Mittal et al., 2018; Wu et al., 2019b). Thus, metabolic interactions are present between breast cancer cells and stromal cells (Figure 3), and glucose metabolism in tumor stromal cells is suggested in the reverse Warburg effect. According to the reverse Warburg effect, aerobic glycolysis occurs in CAFs that are present in the breast cancer stroma. In brief, the reverse Warburg theory describes the glycolysis that occurs in CAFs by ROS, HIF1A, and nuclear factor-κ*B* (NF-κB), resulting in lactate being released from CAFs by MCT4, which is then transported into the tumor cells by MCT1 in breast cancer, creating energy by mitochondrial OXPHOS (Pavlides et al., 2009; Fu et al., 2017; Wilde et al., 2017). Lactate produced by CAFs is transported into the tumor cells as potent nutrients for the TCA cycle, and this lactate can be an important source of energy for cancer cells because lactate is the primary source of carbon for the TCA cycle among circulating metabolites (Hui et al., 2017; Martínez-Reyes and Chandel, 2017). In co-cultural studies of breast cancer cell lines and fibroblasts and studies of human breast cancer tissue, MCT4 was expressed in CAFs, whereas MCT1 was expressed in tumor cells (Whitaker-Menezes et al., 2011; Witkiewicz et al., 2012; Johnson et al., 2017). In a co-cultural study of MCF7 breast cancer cells and normal fibroblasts, culture of MCF7 breast cancer cells alone or fibroblasts alone did not exhibit MCT4 expression, whereas co-culture of MCF7 breast cancer cells and fibroblasts showed MCT4 expression in CAFs. The co-culture with fibroblasts showed MCT1 upregulation in MCF7 breast cancer cells (Whitaker-Menezes et al., 2011). Breast CAFs showed higher expressions of GLUT1 and PDK1 than normal fibroblasts (Pasanen et al., 2016), and the co-cultural study of breast cancer cells and fibroblasts showed an increase in glycolysis and glucose transporter-related genes in CAFs (Ueno et al., 2015). The reverse Warburg effect is not only observed between cancer cells and CAFs but also between hypoxic and oxygenated cancer cells (Sonveaux et al., 2008; Doherty and Cleveland, 2013).

One type of immune cells in the tumor stroma is tumor-associated macrophages (TAMs) that inhibit antitumor immunity in breast cancer, resulting in tumor progression. In general, TAMs exhibit properties of M2 macrophages (Mantovani et al., 2002; Hollmén et al., 2015), and TAMs in hypoxic tumor regions express HIF-1 (Burke et al., 2003), which controls the expression of glycolysis-related genes, including *GLUT1*, *HK2*, *PFFB3*, and *PGK1* (Semenza et al., 1994). Therefore, TAMs in hypoxic tumor environments may utilize glycolysis. In addition, lactate generated in the glycolysis process is an important metabolite, which activates M2 macrophages (Colegio et al., 2014; Chen P. et al., 2017; Mu et al., 2018). In a co-culture study of breast cancer cells and macrophages, G protein-coupled receptor 132 (Gpr132) senses lactate in the tumor environment to transform macrophages into M2-like phenotypes to promote cancer cell adherence, migration, and invasion (Chen P. et al., 2017). In addition, HIF-1α-stabilizing long non-coding RNA (HISLA) is transferred from TAMs to breast cancer cells *via* extracurricular vessel transmission, which increases glycolysis in breast cancer cells (Chen et al., 2019).

## Impact of Glucose Metabolism and Glucose Transporters on Breast Cancer Biology and the Response to Treatment

First, the proliferation of tumor cells requires a lot of energy and a variety of materials are needed to create new tumor cells, which is also true for breast cancer cells. Therefore, glucose metabolism and glucose transporters, which provide energy sources for breast cancer, and PPP, which provides the materials needed for the synthesis of nucleotides, lipids, and non-essential amino acids, play important roles in breast cancer proliferation. Second, glucose metabolism affects the maintenance of epithelial-mesenchymal transition (EMT) and cancer stem cell (CSC) phenotype in breast cancer. Increased glycolysis and PPP by epigenetic silencing of fructose-1,6-biphosphatase can increase NADPH and reduce ROS levels, which enhance EMT and CSC phenotype in basal-like breast cancer (Dong et al., 2013; Schieber and Chandel, 2013). In a breast cancer cell line study, high glucose levels increased glycolytic enzyme, motor protein, and NF-κB levels and glucose uptake, and reduced actin, resulting in EMT phenotype activation (Santos and Hussain, 2020). In addition, HIF-1 activation by hypoxia maintains ROS homeostasis through the glycolytic pathway and serine synthesis pathway, which is important for breast CSC induction (Semenza, 2017). Moreover, glucose metabolism is associated with treatment resistance in breast cancer, where induced glycolysis is observed by AKT/mTOR/HIF-1α axis activation in tamoxifen resistant breast cancer cells, and when HKII is inhibited, tamoxifen sensitivity is recovered (Woo et al., 2015). Increased glycolysis is observed in trastuzumab resistant breast cancer cells, and glycolytic inhibition reduces trastuzumab resistance (Zhao et al., 2011). The expression of PFK-2 is linked to the responsiveness of anticancer drugs such as epirubicin and 5-fluorouacil in breast cancer cells (Benesch et al., 2010; Lin et al., 2015). Chemoresistant TNBC cells exhibit increased glycolysis and lactate permutation (Zhou et al., 2010), and PHGDH expression correlates with the responsiveness of chemotherapy in TNBC cells (Samanta et al., 2016). GLUT is associated with breast cancer metastasis; a proteomic analysis of MDA-MB-231 (metastatic breast cancer cell line) and MCF-10A (normal breast epithelial cell line) showed that one of the three strongest breast cancer-related proteins was GLUT1 (Risha et al., 2020). The GLUT expression showed a difference according to the metastatic sites, and the expression of GLUT1 was the highest in brain metastasis (Kim H. M. et al., 2014). Additionally, GLUT12 plays an important role in tumor growth and metastasis through aerobic glycolysis in TNBC (Shi et al., 2020).

## Clinical Application of Glucose Transporters and Glucose Metabolism in Breast Cancer

As we have seen earlier, glucose transporter expression is high in breast cancer, and glucose metabolism is carried through the glycolytic, serine/glycine, and PPPs that play important roles in tumor growth and progression. Therefore, they may have a variety of clinical applications, especially in imaging diagnosis and targeted therapy.

### Imaging Diagnosis

Positron emission tomography (PET) using ^18^F-fluorodeoxy glucose (FDG), a radioactive analog of glucose, is the representative functional imaging technique based on the principle that tumor cells uptake large amounts of glucose by GLUT *via* the Warburg effect. These PETs are used for tumor staging and treatment response monitoring (Bohndiek and Brindle, 2010). These FDG-PET/CTs are also useful for diagnosis, staging, and treatment evaluation in breast cancer (Groheux et al., 2016; Caresia Aroztegui et al., 2017; Paydary et al., 2019). In addition to FDG-PET/CT, functional imaging based on glucose metabolism can be performed using magnetic resistance spectroscopy (MRS). Multiple metabolites can be simultaneously identified in tumor tissues using MRS, which can analyze labeling patterns using stable isotopic traces, and glucose metabolites can be analyzed using ^13^C-MRS and [^13^C]-labeled glucose to image the glycolysis status. MRS can perform effective metabolic monitoring in breast cancer (Rivenzon-Segal et al., 2002). Breast cancer with different ^13^C-MRS expression patterns show a different glucose metabolism (Grinde et al., 2011). A high-resolution magic angle spinning MRS analysis of metabolites in breast cancer, such as β-glucose, lactate, and glycine, shows good prognosis with reduced concentrations of glycine. The concentration of β-glucose shows a negative correlation with proliferation index (MIB-1), indicating that MR metabolite analysis is valuable in breast cancer prognostication (Sitter et al., 2010).

### Therapeutic Target of Glucose Metabolism and Glucose Transporters

**FIGURE 4:**
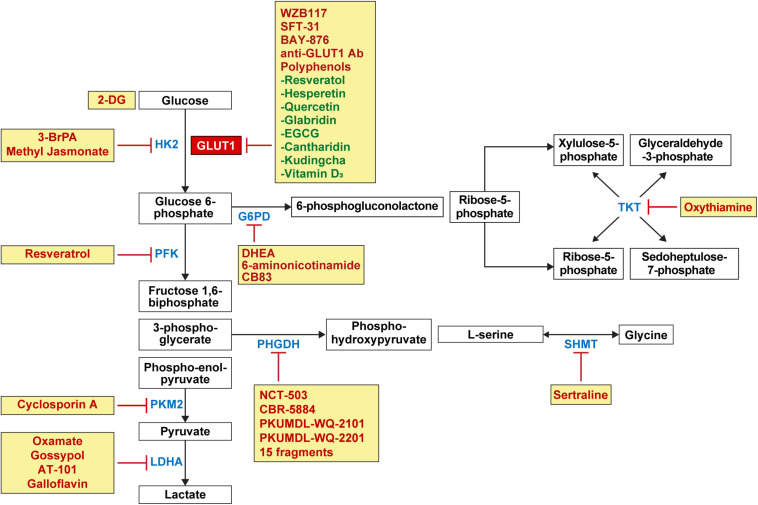
Candidate drugs for the inhibition of glucose metabolism and glucose transporters in breast cancer. Inhibitors for GLUT1 involved in glucose influx in glycolysis include WZB117, SFT-31, BAY-876, anti-GLUT1 antibody, and polyphenols such as resveratrol, hesperetin, quercetin, glabridin, EGCG, cantharidin, kudingcha, and vitamin D_3_. 2-DG competes with glucose for binding GLUT1. Enzyme inhibitors for HK2 involved in glycolysis include 3-BrPA and methyl jasmonate, and resveratrol as PKF inhibitors; cyclosporine A as PKM2 inhibitor; and oxamate, gossypol, AT-101, and galloflavin as LDHA inhibitors. Enzyme inhibitors for G6PD involved in PPP include DHEA, 6-aminonicotinamide, and CB83 and oxythiamine as TKT inhibitor. In the serine and glycine pathway, PHGDH inhibitors include NCT-503, CBR-5884, PKUMDL-WQ-2101, PKUMDL-WQ-2201, and 15 fragments, and sertraline as SHMT inhibitors. HK, hexokinase; PKF, phosphofructokinase; PKM2, pyruvate kinase isozymes M2; LDHA, lactate dehydrogenase A; G6PD, glucose 6-phosphate dehydrogenase; PPP, pentose phosphate pathway; DHEA, dehydroepiandrosterone; TKT, transketolase; PHGDH, phosphoglycerate dehydrogenase; SHMT, serine hydroxymethyltransferase.

The expression of glucose transporters and glucose metabolic enzymes in breast cancer is high; thus, their inhibition can serve as an effective treatment strategy against breast cancer (Figure 4). Various preclinical and clinical studies have been conducted to investigate this implication.

#### GLUT1 Inhibitors

GLUT1 inhibitors—WZB117 and SFT-31—inhibit cell proliferation and promote apoptosis in breast cancer cell lines (Xintaropoulou et al., 2015). WZB117 increases the effectiveness of radiation (Zhao et al., 2016) and anticancer drugs in breast cancer cell lines (Liu et al., 2012; Chen Q. et al., 2017). BAY-876, a selective GLUT1 inhibitor, decreases glucose uptake in TNBC cell lines (Wu et al., 2019a) and 2-deoxy-D-glucose (2-DG), a synthetic non-metabolizable glucose analog, competes with glucose for binding GLUT, which reduces glucose uptake in the MDA-MB-231 TNBC cell line (Amaral et al., 2018). As for 2-DG, there are two different phenomena resulting from the suppression of glycolysis: first, glucose can be deviated to PPP because 2-DG is not metabolized any further after phosphorylation into 2-deoxy-D-glucose-6-phosphate (2-DG-6-P) by HKII (Ralser et al., 2008); and second, 2-DG induces autophagy due to endoplasmic reticulum (ER) stress. Suppression of glycolysis leads to a decreased ATP, by which *N*-linked glycosylation is suppressed and AMPK is activated. AMPK activation and *N*-linked glycosylation lead to ER stress (Xi et al., 2011, 2013). Autophagy promotes tumor growth in the early stage of cancer (Cheong, 2015), maintains tumor survival, and increases metastasis in the advanced stage (Yang et al., 2011). Anti-GLUT1 monoclonal antibody decreases glucose uptake in the MDA-MB-231 TNBC cell line, and decreases cancer cell proliferation and promotes apoptosis in MCF-1 and T47D breast cancer cell lines (Rastogi et al., 2007). Polyphenols, a huge family of natural compounds found in plants or food, is one category of the GLUT1 inhibitors (Williamson, 2017) that shows an anti-tumoral effect against various cancers including breast cancer. The anti-tumoral mechanism of polyphenols against breast cancer includes increased apoptosis, cell cycle arrest, enhanced autophagy, decreased angiogenesis, anti-inflammatory effect, blockade for estrogen, aromatase modulation, altered redox balance, and inhibition of the HER-2 pathway (Mocanu et al., 2015; Losada-Echeberría et al., 2017). Polyphenols inhibiting GLUT1 in breast cancer are as follows: Resveratol suppresses glucose uptake in T-47D cell line by reducing GLUT1 protein level (Jung et al., 2013), and hesperetin suppresses glucose uptake by decreasing GLUT 1 mRNA and protein levels (Yang et al., 2013). Quercetin decreases the glucose uptake in MCF-7 and MDA-MB-231 by reducing GLUT1 protein level (Jia L. et al., 2018), as does glabridin in MDA-MB-231 (Li et al., 2019). Epigallocatechin-3-gallate (EGCG) decreases the glucose and lactate levels in cancer cells by reducing GLUT1 mRNA levels in 4T1 cell line (Wei et al., 2018), and cantharidin suppresses metastasis by inhibiting glucose uptake and lactate production through decreasing GLUT1 protein level in MCF-7 and MDA-MB-231 (Pan et al., 2019). Kudingcha, one of the Ligustrum robustum species, inhibit cancer proliferation through decreasing GLUT1 protein level in MDA-MB-231 and HCC1806 (Zhu et al., 2020). Vitamin D_3_ decreases glucose uptake by decreasing GLUT1 mRNA and protein levels in MCF-7 and MDA-MB-231 (Santos et al., 2018).

#### Glucose Metabolic Enzyme Inhibitors

First, 3-bromopyruvate (3-BrPA), an inhibitor of hexokinase, causes apoptosis in MDA-MB-231 breast cancer cell line (Liu et al., 2014; Chen et al., 2018) and increases the response to daunorubicin (Liu et al., 2015) and tamoxifen (Attia et al., 2015) in breast cancer. Methyl jasmonate, another hexokinase inhibitor, caused a decrease in tumor volume in mice bearing 4T1 breast cancer cell line (Yousefi et al., 2020). Resveratrol, an inhibitor of PFK, decreases the cell viability and glucose consumption in MCF-7 breast cancer cell line (Gomez et al., 2013). Cyclosporin A, an immunosuppressive agent, inhibits the expression and activity of PKM2 in breast cancer cell lines (MCF-7, MDA-MB-435, and MDA-MB-231) and causes tumor cell death by reducing cell viability (Jiang K. et al., 2012). Cyclosporin A also maintains mitochondrial function by suppressing mitochondrial permeability transition pore (Halestrap et al., 1997; Mishra et al., 2019). When oxamate, an LDH inhibitor, is administered in conjunction with doxorubicin and metformin, it causes a rapid tumor growth inhibition in the xenograft model using human MDA-MB-231 TNBC cell line (García-Castillo et al., 2017). When paclitaxel and oxamate are administered together, they induce an effective killing of paclitaxel-resident TNBC cells (Zhou et al., 2010). Gossypol, a lipid soluble polyphenolic compound, exhibits antitumor effects by inhibiting glycolysis through LDH isoenzyme type 5 inhibition (Coyle et al., 1994). Gossypol causes anti-proliferative activity and apoptosis in breast cancer cells (Gilbert et al., 1995; Ye et al., 2010; Messeha et al., 2019), and when R-(-)-gossypol (AT-101) is administered in conjunction with trastuzumab in HER-2 positive breast cancer cell line, it causes synergistic cytotoxicity and apoptosis (Bulut et al., 2020). Galloflavin, an LDHA inhibitor, induces cell death in MDA-MB-231 cell lines and acquired tamoxifen resistance MCF-7 breast cancer cell lines (Farabegoli et al., 2012).

Serine and glycine pathway inhibitors can be used for the management of tumors that use serine and glycine metabolism and for treatment of tumors showing recurrence and treatment resistance. PHGDH inhibitors—NCT-503 and CBR-5884—are both allosteric PHGDH inhibitors; NCT-503 binds to the near substrate-binding pockets; and CBR-5884 hinders PHGD holigomerization (Mullarky et al., 2016; Pacold et al., 2016). NCT-503 inhibits tumor growth in PHGDH-amplified breast cancer xenografts (Pacold et al., 2016), and CBR-5884 inhibits tumor cell proliferation in high PHGDH-expressing breast cancer cell lines (MDA-MB-468, MDA-MB-436, HCC70, and Hs578T) (Mullarky et al., 2016). PKUMDL-WQ-2101 and PKUMDL-WQ-2201, which are allosteric PHGDH inhibitors, show an antitumor activity in PHGDH-amplified breast cancer cell lines (MDA-MB-468 and HCC70) (Wang et al., 2017). An NAD-competitive PHGDH inhibitor, 15 fragments, reduces cell proliferation in *PHGDH*-amplified breast cancer cell line (MDA-MB-468) (Unterlass et al., 2018). Sertraline, an antidepressant, is a selective serotonin reuptake inhibitor (SSRI) class (MacQueen et al., 2001), but it also works as a competitive dual SHMT1/2 inhibitor, reducing the cell growth in serine/glycine synthesis-addicted breast cancer cell line (MDA-MB-468) and decreasing the tumor growth in a mouse xenograft study (Geeraerts et al., 2021b).

G6PD, one of the important enzymes in PPP, has a potent non-competitive inhibitor, dehydroepiandrosterone (DHEA), which is an adrenal cortical steroid. DHEA inhibits the growth and migration of breast cancer cell lines (MCF-7, MDA-MB-231, and Hs578T) (López-Marure et al., 2011). DHEA can bind estrogen or androgen receptors because it is metabolized to estrogen or androgen (Labrie et al., 2001), however, the suppression of MCT-7 cell line growth by DHEA is reported to be independent of estrogen or androgen receptors (Gayosso et al., 2006). 6-aminonicotinamide, a G6PD inhibitor, can decrease mammosphere formation and aldehyde dehydrogenase (ALDH) activity when given with DHEA in breast cancer stem-like cells that show high PPP activity (Debeb et al., 2016). CB83, another G6PD inhibitor, can inhibit growth of MCF10-AT1 breast cancer cell line (Preuss et al., 2013). Oxythiamine, an inhibitor of TKT, also increases the response of breast cancer cells to doxorubicin or docetaxel (Tseng et al., 2018).

## Conclusion

Because of the high expressions of GLUT-1 and the enzymes involved in glucose metabolism, tumor cells in breast cancer, as in other tumors, are provided with energy through glucose metabolism. There are several characteristic factors to consider in the glucose metabolism of breast cancer. Because breast cancer is heterogeneous, inter- and intratumoral heterogeneity is also seen in glucose metabolism. First, glucose metabolic activity is different among the molecular subtypes, especially in TNBC, which shows an increased glycolytic phenotype (Wang et al., 2020). According to the traditional Warburg theory, tumors showing aerobic glycolysis are suggested to exhibit a decreased mitochondrial OXPHOS; however, TNBC with a high metabolic activity shows both enhanced glycolysis and sustained mitochondrial OXPHOS (Park et al., 2016; Lanning et al., 2017; Luo et al., 2018; Jia et al., 2019). Luminal type breast cancer rely more on OXPHOS than glycolysis compared to TNBC (Pelicano et al., 2014). It also presents metabolic switches between glycolysis and OXPHOS during cancer progression (Levine and Puzio-Kuter, 2010; Jia D. et al., 2018; Lai et al., 2020; Moldogazieva et al., 2020). Therefore, metabolic intratumoral heterogeneity is exhibited in breast cancer, showing different glycolytic activities depending on the tumor cell type. Second, there is a metabolic interaction between tumor cells and the surrounding stromal cells in breast cancer. Breast cancer is a typical tumor that contains various stromal cells, the main components of which are CAFs, CAAs, and immune cells. Metabolic interactions exist between breast cancer cells and stromal cells; especially according to the reverse Warburg theory, lactates produced by glycolysis in CAFs enter tumor cells and produce energy through OXPHOS. Among the immune cells, B-cells and NK cells use glycolysis, and tumor-associated neutrophils use glycolysis and PPP, allowing a metabolic competition with the tumor cells. Third, unlike in other tumors, CAAs are stromal cells that are specifically present in breast cancer, and previous studies suggest that β-oxidation in tumor cells is primarily studied through the lipid transfer between CAAs and tumor cells. As the glucose metabolic interaction between CAAs and tumor cells is rarely studied in breast cancer, it requires further study. Metabolic interactions between tumor cells and stromal cells in these breast cancer cases are also reported to be affected by cancer phenotypes (Brauer et al., 2013), which may require further research on the metabolic cross-talk between the cancer cells and stromal cells according to the molecular subtype of breast cancer. Fourth, breast cancer shows differential metabolic features depending on the stage and metastatic site. In order for the tumor to progress into distant metastasis, multiple and complex processes, such as intravasation, survival in blood stream, and extravasation, must be accomplished during this process, and the hurdles, such as anchorage independent survival and tumor cell proliferation in foreign microenvironment, should be overcome. One way to overcome this challenge is metabolic reprogramming. Breast cancer shows metabolic differences between the primary and metastatic tumors (Chen et al., 2007; LeBleu et al., 2014; Dupuy et al., 2015; Simões et al., 2015; Andrzejewski et al., 2017), and breast cancer does not rely on a single metabolic pathway, but uses multiple metabolic pathways. Highly metastatic 4T1 cells show increased glycolysis and OXPHOS compared to non-metastatic 67NR breast cancer cells (Simões et al., 2015). The most common metastatic sites are the brain, bone, lung, and liver, which exhibit differential metabolic features owing to different microenvironments. Liver metastatic breast cancer demonstrates increased glycolytic pathways compared to bone and lung metastatic breast cancer (Dupuy et al., 2015), whereas brain metastatic breast cancer shows increased glycolysis and PPP compared to bone metastatic breast cancer (Chen et al., 2007). As a result of the above characteristics of glucose metabolism in breast cancer, further studies are needed to consider tumor imaging using glucose metabolism and glucose metabolic markers as treatment targets. In addition, because glucose metabolism is associated with resistance to anticancer drugs or targeted treatments in breast cancer, glucose metabolic inhibitors can also be considered for a combined therapy with conventional treatments.

## Author Contributions

ES and JK: writing—original draft, investigation, and writing—review and editing. Both authors contributed to the article and approved the submitted version.

## Conflict of Interest

The authors declare that the research was conducted in the absence of any commercial or financial relationships that could be construed as a potential conflict of interest.

## Publisher’s Note

All claims expressed in this article are solely those of the authors and do not necessarily represent those of their affiliated organizations, or those of the publisher, the editors and the reviewers. Any product that may be evaluated in this article, or claim that may be made by its manufacturer, is not guaranteed or endorsed by the publisher.

## Abbreviations

HK2: hexokinase II
PFK: phosphofructokinase
PKM2: pyruvate kinase isozymes M2
LDHA: lactate dehydrogenase A
G6PD: glucose 6-phosphate dehydrogenase
6PGD: 6-phosphogluconate dehydrogenase
RPE: ribulose-5-phosphate epimerase
RPI: ribose 5-phosphate isomerase
TKT: transketolase
TALDO: transaldolase
PHGDH: phosphoglycerate dehydrogenase
PSAT1: phosphohydroxythreonine aminotransferase
PSPH: phosphoserine phosphatase
SHMT: serine hydroxymethyltransferase
GLDC: glycine decarboxylase
CAF: cancer-associated fibroblast
HIF: hypoxia-inducible factor
MCT: Monocarboxylate transporter
OXPHOS: oxidative phosphorylation
TAM: tumor-associated macrophage
PPP: pentose phosphate pathway
DHEA: dehydroepiandrosterone
PHGDH: phosphoglycerate dehydrogenase
ROS: reactive oxygen species
EMT: epithelial-mesenchymal transition
CSC: cancer stem cell
NADPH: nicotinamide adenine dinucleotide phosphate
6PGDH: 6-phosphogluconate dehydrogenase
PI3K: phosphoinositide 3-kinase
mTOR: mammalian target of rapamycin
AMPK: AMP-activated protein kinase
VEGF: vascular endothelial growth factor
BMI: body mass index
E2: 17 beta-estradiol
IGF: insulin-like growth factor.
